# Biomechanical modeling and imaging for knee osteoarthritis – is there a role for AI?

**DOI:** 10.1016/j.ostima.2024.100182

**Published:** 2024-05-21

**Authors:** Mika E. Mononen, Mikael J. Turunen, Lauri Stenroth, Simo Saarakkala, Mikael Boesen

**Affiliations:** a Department of Technical Physics, University of Eastern Finland, Kuopio, Finland; b Science Service Center, Kuopio University Hospital, The Wellbeing Services County of North Savo, Kuopio, Finland; c Research Unit of Health Sciences and Technology, University of Oulu, Oulu, Finland; d Department of Diagnostic Radiology, Oulu University Hospital, Oulu, Finland; e Department of Radiology, Copenhagen University Hospital Bispebjerg and Frederiksberg, and Institute of Clinical Medicine, University of Copenhagen, Copenhagen, Denmark

**Keywords:** Osteoarthritis, Prediction, Finite element analysis, Machine learning, Deep learning

## Abstract

**Objective:**

This mini review aims to assess the latest advancements in the field of osteoarthritis (OA) research, particularly focusing on the early detection and prediction of disease progression through the use of advanced imaging technologies utilizing biomechanical modeling and artificial intelligence (AI).

**Design:**

The review consolidates and discusses findings from studies that utilize biomechanical modeling and/or machine learning algorithms to identify pathological changes in joint tissues indicative of OA or prediction of disease progression. It also briefly reviews future research and how these methods could be used as a part of OA management.

**Results:**

AI algorithms have proven highly effective in recognizing the subtle changes in joint tissues associated with OA and in identifying patients at high risk for the disease. While these automated tools facilitate early diagnosis, they typically do not provide personalized intervention strategies to prevent disease progression. AI-enhanced biomechanical modeling has the potential to simulate the effects of various conservative interventions (e.g., weight management, optimal footwear, and gait retraining) on slowing OA progression, which could be pivotal for patient engagement and preventive care.

**Conclusions:**

The integration of AI with biomechanical modeling holds significant promise for enhancing the management of OA by not only predicting disease onset and progression but also by enabling personalized intervention plans. Future research should focus on the development of these models to include personalized, preventive strategies that could effectively engage patients and potentially delay or prevent the onset of OA. This approach could revolutionize patient care by making early, targeted intervention feasible.

## 1 Introduction

In a typical clinical case, a patient comes to a physician's office because of knee pain. Subsequently, after routine clinical examination, the physician often writes a referral for imaging examination, primarily to knee radiography. Whether the diagnosis is a mild injury, early osteoarthritis (OA), or a sprain, the patient leaves the office with only generic treatment instructions, such as lose weight (if obese), stay physically active, and use medications for pain. Current general guidelines, however, focus only on the treatment of current symptoms, whereas a presumably more beneficial approach would give the patient easily understandable information on the condition of the knee and possible future risk of OA, based on quantitative data. Current practice is a little paradoxical as there have tremendous developments in the diagnosis of early OA with artificial intelligence (AI) algorithms and methods and yet our advice to the patient is still generic.

Several grading systems have been developed to determine the integrity of the knee joint structures based on X-ray or magnetic resonance imaging: Kellgren-Lawrence grade (KL), MRI Osteoarthritis Knee Score (MOAKS), Whole-Organ Magnetic Resonance Imaging Score (WORMS). These grading systems are suitable for defining the current OA status of the knee, but they do not directly help clinicians to provide patients with quantitative information about their risk of developing OA, or how conservative interventions, such as weight loss, muscle strengthening, dietary interventions, selecting an optimal type of shoe, or gait re-training, could reduce the risk of progression. The effect of the proposed intervention on the prognosis would be highly important, as seeing the data visualized, has been shown to be a key factor in patient engagement [1].

**Fig. 1:**
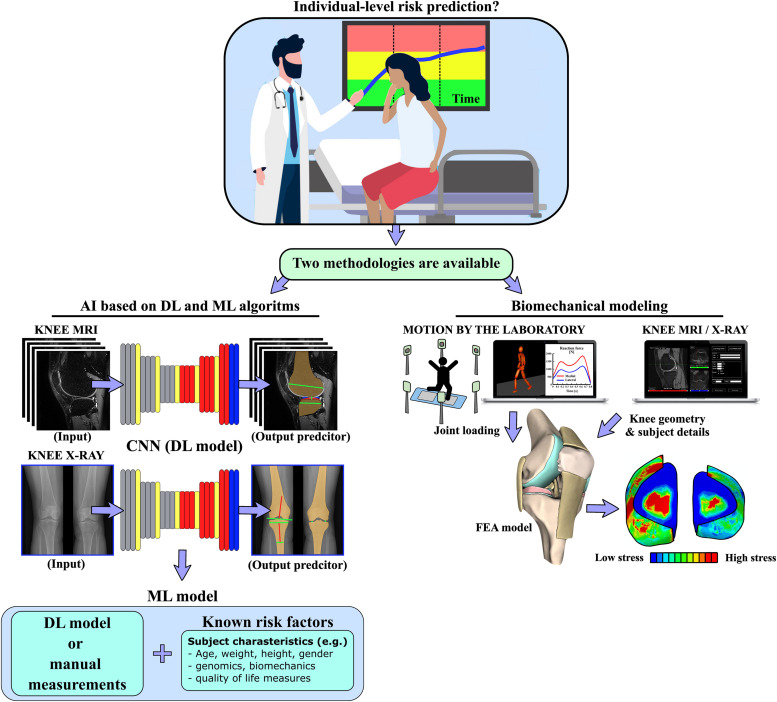
A schematic presentation of how current methodologies for AI and FEA-based models differ when predicting individual-level risk for the onset and progression of knee OA.

In the current literature, two methodologically different approaches to predict individual-level risk for the onset and progression of knee OA have been introduced. The first one can be called *biomechanical modeling*, i.e., the prediction is given by the simulated mechanical responses of the joint tissues [2]. The second can be called *AI*, which is based on machine learning (ML) and/or deep learning (DL) algorithms [[3], [4]] without an underlying physical model (Fig. 1). Common to both approaches is that they use data from clinical imaging, and their predictive abilities have been validated against quantitative values of OA severity such as KL grade, or cartilage thickness loss [[5], [6]]. The diversity of the target variables, however, can create challenges in interpreting the results between different approaches because there is no consensus on how the clinical data should be use to quantify the severity of the OA. Some researchers rely heavily on X-ray based KL grade, while others rely on cartilage thinning to validate their models.

In this mini review, we present selected recent studies using biomechanical modeling and AI based approaches to predict individual-level risk for the onset and progression of knee OA. The advantages and disadvantages of these methods will be presented briefly, followed by an overview of the possibilities and potential of merging the two approaches to create a scalable tool for clinical use to engage patients to reduce their personal risk for OA.

## 2 Osteoarthritis prediction

### 2.1 Biomechanical modeling

In biomechanical knee joint models, the modeled geometry of bone, cartilage, meniscus, ligament, and tendon tissues, depending on the type of model, is based on clinical imaging data. The joint level model is typically simulated while walking [2]. This is justified since walking is the most common physical activity among all people [7]. If we assume that the joint loading conditions and material properties for joint tissues are accurate, the predicted force distributions in the knee can be considered valid for simulation. Unfortunately, this is not the case as there are no validated methods for determining patient-specific material properties of joint tissues from clinical imaging data. Secondly, there are many sophisticated methods for estimating the loading conditions at the knee level [8], but these methods suffer from measurement errors and unverifiable assumptions. Moreover, the methods require extensive experimental and data processing efforts, which have prevented them from being applied in large studies with years of follow-up. Therefore, the capability and utility of biomechanical modeling-based predictions of knee OA have not been properly validated.

Although biomechanical modeling has its shortcomings and simulations, usually taking hours for each simulated knee, an advantage is that the results do not depend on training data, as each individual biomechanical model is based solely on the data collected from that particular subject. The models are also easily understandable as they are physics-based and allow simulation of hypothetical scenarios such as the effects of weight loss [2]. If the current methodological challenges associated with biomechanical modeling and simulation of knee function can be overcome, a recently introduced atlas-based finite element analysis (FEA) approach could be a clinically applicable solution, as it requires only imaging data (geometry of knee and cartilage thickness) and simple subject characteristics (age, weight). Briefly, this method uses an existing template finite element model, and its loading conditions are scaled based on the anatomical dimensions of knee and weight. Predicted cartilage degradation is calculated by post-processing the simulated tensile stress distributions, considering the average failure properties of the cartilage tissue as a function of ageing [[9], [10]]. This kind of a simulation is easy to scale to be used by other researchers as it does not require previous experience on FEA tools and even though it is simplified, the approach has already shown potential in predicting the progression of OA [12].

### 2.2 Machine learning (ML) based algorithms

The current main trend in the prediction of OA progression focuses mainly on developing quantitative methods and models to separate healthy from OA patients utilizing ML and, more specifically, DL algorithms [[5], [6], [11], [12]]. Generally, these models are trained to classify patients into different degrees of knee OA after a given time period with different prognostic variables. In many of these models, image features are typically extracted by trained DL models using a convolutional neural network (CNN) [[5], [6], [11]], but features can be extracted manually [12]. Usually, the extracted features are related to the articular cartilage, but in a study by Almhdie-Imjabban et al. [6], the authors focused on bone and extracted 64 different features from the bone beneath the cartilage. Subsequently, image-based features were combined with information obtained from subject characteristics and physical performance measurements or health related questionnaires using various ML/DL approaches from simple regression [6] to more complex DenseNet [5]. Although models are built from readily available data using automatic image feature recognition enabled by DL algorithms, many models also use the clinical information from WOMAC or KOOS questionnaires or physical performance tests as predictor variables [[5], [11], [12]]. This approach can increase the number of possible predictor parameters up to or more than 100 [12]. The large number of required predictor variables can be a major problem for a method to be suitable as a clinical tool, as collecting all the necessary variables from patients can be impractical.

## 3 Summary and discussion

The AUC (Area Under the Curve) - ROC (Receiver Operating Characteristics) curve is a fundamental metric for evaluating the performance of a diagnostic test [13]. AUC-ROC analysis is also used when evaluating and validating different methods for predicting OA progression. The differences in classification between FEA and AI based models discussed in this mini-review article are shown in Table 1. In general, the published ML/DL based methods are more accurate at classification than the published FEA based methods. As noted earlier, however, FEA can be used to generate models with higher patient-specificity than the models considered in this work, but the current lack of methodology for determination of patient-specific tissue material properties and large datasets with patient-specific loading inputs limits the validation of more personalized FEA models. It must be noted that although the reported AUC values may look promising for the ML and DL based approaches, the trained models may not perform as well in clinical practice. A recent article strongly suggests that current reported AI models are still highly dependent on the data on which they are trained [14] and, thus, probably perform worse when used with novel data. On the other hand, this can also be the case with FEA models, as they have been validated using patient data with specific inclusion criteria, which again may limit their applicability to other populations.

**Table 1:** Overview of the reported prediction performances in the articles reviewed.

Reference	Modality	Image feature extraction *	Target variable ⁎⁎	Training data	Prediction model	Performance (AUC)
[5]	X-ray	CNN	Joint space loss	*N* = 4447	YOLO & DenseNet	0.86
[6]	X-Ray	CNN	Joint space loss	*N* = 2571	Regression	0.75 - 0.81
[9]	MRI	Manual	KL	n/a	FEA	0.68
[10]	MRI	Manual	KL	n/a	FEA	0.73
[11]	X-ray	CNN	Change in KL	*N* = 4928	Gradient Boosting Machine	0.79
[12]	MRI	Semi-automatic	KL	*N* = 1044	XGBoost	0.77 - 0.79

⁎ CNN = Convolution Neural Network.

⁎⁎ KL = Kellgren-Lawarence grade.

Another important point to consider is that the comparison of the AUC values alone is insufficient for judging whether one model is better than another, since the distribution of the training data might vary between different studies, and it is well-known that AUC values are dependent on the balance/imbalance in the datasets [15]. Although repeated cross-validation can improve the confidence of the reported AUC value [16], it does not eliminate the training data imbalance problem. Therefore, other metrics such as a weighted F1 score that accounts for imbalances in class distribution [17] would be a better measure to assess the performance of the trained models, but the problem with current studies is that this parameter is less likely to be reported. Furthermore, the target variables and time periods for prediction may differ between the models. For example, in the Guan et al. study [5], progressive OA was defined as joint space narrowing by more than 0.7 mm, whereas other studies usually define progressive/severe osteoarthritis as changes in the KL grade [[9], [10], [11], [12]]. It would also likely be beneficial to normalize joint space narrowing to the initial joint space width rather than using absolute values. For example, a 1 mm reduction in joint space in patients with initial joint space of 1 cm could be clinically non-significant although exceeding the previously used threshold of progressive OA. Thinning cartilage, or joint space narrowing, is a normal age-related change, even when there are no other symptoms, but this process may be speeded up if subjected to risk factors like trauma, obesity, hard physical work, or potentially halted/slowed after treatment [[18], [19]]. It would, therefore, be preferable to use threshold values that are linked to the knee OA severity, like KL grades.

When considering the final use of ML/DL models using questionnaire-based health data models in clinical or commercial settings, a major challenge is to motivate patients to respond honestly and without bias. It is well known that self-reported levels of pain vary greatly between individuals depending, e.g., on pain sensitization and other psychosocial factors. Furthermore, some may exaggerate or, on the contrary, underestimate their symptoms or performance. This is influenced by many factors like the person's own goals and what the results of the prediction models will be used for. For example, patients may respond differently if the results will be used to estimate their insurance premiums, rather than if the results are to determine their access to care. Therefore, clinically applicable models should use a set of objective rather than subjective predictor variables such as data obtained directly from clinical images, subject characteristics (e.g., age, weight and sex), quantitative sensory testing, and data from previous clinical visits such as history of joint injuries.

Clinical imaging data is the foundation of both approaches and cannot be substituted. Although the methods look very promising in terms of the reported AUC values, both approaches still need to be improved and validated with independent datasets before any of the published methods can be applied clinically.

## 4 Future aspects

### 4.1 Biomechanics merged with AI

Considering these two methodologically different approaches to predicting individual-level risk for the onset and progression of knee OA, many researchers are asking the question: What is the best way to use the best aspects of FEA and ML/DL based models? Unfortunately, there is currently no definitive answer to this question. Still, ML/DL based algorithms and statistical shape models offer new ways to generate model inputs that are needed for biomechanical FEA models. For instance, personalized knee joint loading conditions can be predicted through the use of statistical shape models based on MRI data or with ML models based on the subject characteristics and a few other simple physical measures that are usually reported in many randomized controlled trials with several years of follow-up. The application of such an approach in the FEA studies reviewed here is anticipated. Furthermore, portable, out-of-lab sensing solutions with wearable sensors or video recording data can also be considered as a solution for subject-specific loading conditions. The limitation here is the lack of strong follow-up data for validating the model's performance in predicting progression. It could be hypothesized that a more accurate estimate of personalized loading conditions in FEA would lead to better prediction of personalized risk. In the future, ML/DL based methods will enable the prediction of FEA-based results. It should be noted, however, that these solutions cannot outperform the traditional FEA workflow. One interesting aspect, which has not yet been studied, would be to investigate whether, and how well, ML/DL based models could improve FEA based predictions and vice versa. We believe that these issues are ripe for clarification in future studies of OA prediction.

### 4.2 Patient engagement

In terms of treatment and engagement of patients and improvement in the quality of life, the most critical aspect should be to focus on the final purpose of their use and whether the developed model serves the matter in question, rather than developing forecast models. In other words, the prediction should lead to an action that reduces the risk of, or rate of OA progression. As far as we know, only one study has provided a visual user interface merged with a prediction algorithm [20]. This prediction algorithm is based on statistical modeling of the risk of developing OA over different periods. The biggest shortcoming of such a model is that it only provides a generalized prognosis and, thus, cannot provide a feasible estimate of the effects of different conservative interventions, such as weight loss. These types of models have only a minor effect on the predicted risk and do not result in lifestyle adaptation.

What then would be the optimal way to engage patients in conservative treatments to reduce their risk of OA and work towards a healthier life? Along with the risk predicted by the status of the patient, predictive models can be used to assess the effect of conservative treatments, such as weight loss, on risk. It has been demonstrated that individualized, easily interpretable visual data provided by the predictive models can help to engage patients in making lifestyle changes that can decrease their risk of serious disease [4]. In the future, management of knee OA may also be possible by applying some of the approaches reviewed here. These approaches should be based on real-life data and validated models, but unfortunately, this information is not yet available. Therefore, randomized controlled trials with different visualization interfaces based on validated model results are needed to study patient engagement. Will this approach lead patients to take seriously conservative treatment options like weight loss, muscle strengthening, dietary interventions, shoe type, and/or gait re-training? It will still take a good deal of work to understand whether this approach will actually lead to better outcomes.

## Funding sources

The research leading to these results has received funding from the 10.13039/501100002341Research Council of Finland (grants 324994, 328920, 352666, 332915) and the Sigrid Juselius Foundation (grants 230123, 240130).

## Role of the funding sources

The funding sources had no role in the study design, collection, analysis, or interpretation of data; in the writing of the manuscript; or in the decision to submit the manuscript for publication.

## CRediT authorship contribution statement

**Mika E. Mononen:** Writing – original draft, Writing – review & editing. **Mikael J. Turunen:** Writing – original draft, Writing – review & editing. **Lauri Stenroth:** Writing – original draft, Writing – review & editing. **Simo Saarakkala:** Writing – original draft, Writing – review & editing. **Mikael Boesen:** Writing – original draft, Writing – review & editing.

## Declaration of competing interest

Mika E. Mononen and Mikael J. Turunen own shares in *Aikoa Technologies Oy*.

Simo Saarakkala currently acts as the Associate Editor of Osteoarthritis and Cartilage Open journal.
